# Development of a Signature Based on Eight Metastatic-Related Genes for Prognosis of GC Patients

**DOI:** 10.1007/s12033-023-00671-9

**Published:** 2023-02-15

**Authors:** Fanjing Shang, Yafei Wang, Zixu Shi, Zhidong Deng, Jianwen Ma

**Affiliations:** Department of General Surgery, People’s Hospital of Ningxia Hui Autonomous Region, No. 301 Zhengyuan North Road, Jinfeng District, Yinchuan, 750001 Ningxia China

**Keywords:** Gastric cancer, Prognosis, Metastasis-related genes, Immune, Risk Score

## Abstract

Gastric cancer (GC) has been a common tumor type with high mortality. Distal metastasis is one of the main causes of death in GC patients, which is also related to poor prognosis. The mRNA profiles and clinical information of GC patients were downloaded from The Cancer Genome Atlas and Gene Expression Omnibus databases. Univariate Cox and LASSO Cox analyses were used to screen the optimal metastasis-related genes (MRGs) to establish a prognostic Risk Score model for GC patients. The nomogram was used to visualize the Risk Score and predict the 1-, 3-, 5-year survival rate. The immune cell infiltration was analyzed by CIBERSORT and the ratio of immune–stromal component was calculated by the ESTIMATE algorithm. A total of 142 differentially expressed genes were identified between metastatic and non-metastatic GC samples. The optimal 8 genes, comprising GAMT (guanidinoacetate N-methyltransferase), ABCB5 (ATP-binding cassette subfamily B member 5), ITIH3 (inter-alpha-trypsin inhibitor heavy chain 3), GDF3 (growth differentiation factor 3), VSTM2L (V-set and transmembrane domain-containing 2 like), CIDEA (cell death inducing DFFA like effector a), NPTX1 (neuronal pentraxin-1), and UMOD (uromodulin), were further screened to establish a prognostic Risk Score, which proved to be an independent prognostic factor. Patients in high-risk group had a poor prognosis. There were significant differences in the proportion of 11 tumor-infiltrating immune cells between high-risk and low-risk subgroups. In addition, the StromalScore, ImmuneScore, and ESTIMATEScore in high-risk group were higher than those in low-risk group, indicating that the tumor microenvironment of the high-risk group was more complex. A Risk Score model based on eight metastasis-related genes could clearly distinguish the prognosis of GC patients. The poor prognosis of patients with high-Risk Score might be associated with the complex tumor microenvironments.

**Supplementary Information:**

The online version contains supplementary material available at 10.1007/s12033-023-00671-9.

## Background

Gastric cancer (GC), accounting for about 6% of cancers worldwide, is the second leading cause of cancer-related death and the fifth most commonly diagnosed malignancy in the world [1, 2]. According to the latest global cancer statistics, more than 1,089,103 new cases and 768,793 deaths of GC in 2020 has been estimated [3]. The occurrence and progression of GC are multi-step and multifactorial processes, involving the risk factors, including *Helicobacter pylori* infection, tobacco smoking, and dietary habits [4, 5]. Moreover, during the early stage of GC, there are only a few non-specific mild symptoms, such as upper discomfort and belching, which are difficult to be distinguished from chronic gastritis and chronic gastric ulcer, thereby resulting in easily overlooked aspect in the diagnosis. Since the early stage of GC were asymptomatic or mild, it only showed a few non-specific symptoms, such as upper discomfort and belching, which was similar with chronic gastritis and chronic gastric ulcer, resulting in easy to be ignored in diagnosis [6, 7]. Despite surgical treatment and targeted therapy have made great progress in recent years, the treatment options of GC are still relatively limited, meanwhile the ideal prognosis has not been achieved basing on the current treatments [8]. In addition, patients are often diagnosed with GC at an advanced stage with poor prognosis, accompanied by metastasis, including hepatic metastases and peritoneal dissemination, which is one of the main causes of death in GC patients, and the 5-year survival rate is reduced to 20% [9–11]. Therefore, it is necessary to further explore the deepening mechanism behind GC progression and prognosis, in order to provide better treatment options for patients.

Current studies have suggested that tumor metastasis is a complex process involving multi-gene regulation and multi-step development [12]. Compared with primary tumors, metastatic tumors exhibit poor responses to radiotherapy, chemotherapy, and targeted therapy. More than 90% of cancer patients die from tumor metastasis [13]. Recently, metastasis-related genes have been considered as effective prognostic indicators and widely concerned in a variety of tumors. For example, Yao et al. have showed that MMP (matrix metallopeptidase)-2 and MMP-9, as key genes regulating the degradation of type IV collagen, are able to affect the initial invasion and lymphatic metastasis of tumors and could predict the prognosis of GC patients [14]. Xu et al. have identified four metastasis-related genes in pancreatic cancer, and their low expression levels indicate a poor prognosis [15]. STMN2 has been indicated to be differentially expressed in metastatic and non-metastatic samples of serous ovarian cancer, and high level of STMN2 is associated with poor prognosis of patients [16]. Moreover, several metastasis-related genes associating with prognosis have also been reported in GC, for instance TCF7L2 [17]. Furthermore, the prognostic model based on multiple genes typically exhibits better performance and reliability compared with single gene biomarker. More recently, Tian et al. have demonstrated a metabolism-related signature that could predict the peritoneal metastasis of GC patients [18], while their work has not indicated the directly prognostic role in GC. To the best of our knowledge, the prognostic model comprising multiple metastasis-related genes has been rarely reported in GC.

Accordingly, in our study, the predominant purpose is to explore the prognostic role of metastasis-related genes in GC patients. Herein, a Risk Score model based on 8 metastasis-related genes was constructed to predict the prognosis of GC patients. Our findings provided more novel reference to evaluate the prognosis of GC patients.

## Materials and Methods

### Data Source

Gene expression profiles and clinical data with complete survival information of 342 metastatic and non-metastatic GC patients were obtained from The Cancer Genome Atlas (TCGA) database (https://tcga-data.nci.nih.gov/tcga). Clinical information details of 342 patients are shown in Table 1. In addition, 433 GC samples’ data in GSE84437 were downloaded from Gene Expression Omnibus (GEO) database (https://www.ncbi.nlm.nih.gov/geo/). The gene expression data in GSE84437 were measured using an Illumina HumanHT-12 V3.0 expression beadchip platform.

**Table 1:** Clinicopathological characteristics of GC samples from TCGA database

Parameters	OS Status		x^2^	*P*-value
	Alive (*N* = 199)	Dead (*N* = 143)		
Age (Mean ± SD)	67.02 ± 13.69	66.79 ± 13.68	0.11863	0.7305
Gender			2.2207	0.1362
Female	78	44		
Male	121	99		
Pathologic stage			20.614	0.0003776
i	34	11		
ii	72	35		
iii	77	66		
iv	12	21		
Unknown	5	10		
Grade			2.9204	0.4041
G1	7	2		
G2	75	46		
G3	112	91		
GX	5	4		

### Differential Genes Expression Analysis

The limma package of R software (version 3.5.2, the same below) [19] was applied to identify differentially expressed genes (DEGs). The significant DEGs were screened using the threshold of FDR ≤ 0.05 and Fold Change (FC) > 2.

### Functional Enrichment Analysis

Gene ontology (GO) terms and Kyoto Encyclopedia of Genes and Genomes (KEGG) pathways enrichment analyses were applied on the DEGs using clusterProfiler package of R software (*p* < 0.05 was considered significantly enriched, adjusted by Benjamini and Hochberg method) [20].

### LASSO Cox Regression Analysis

Univariate Cox regression analysis was performed on GC samples based on gene expression values, and *p* < 0.01 was used as threshold to screen genes significantly correlated with prognosis. Then LASSO Cox regression analysis was used to further screen optimal prognostic genes using glmnet of R software [21]. The Risk Score is established by the optimal genes and calculated using the following formula:

$$ {\text{Risk}}\,{\text{Score}} = \sum\limits_{{{\text{i}} = 1}}^{{\text{n}}} {{\text{Coef}}_{{\text{i}}} } *{\text{X}}_{{\text{i}}} , $$

where Coef_i_ is the Risk coefficient of each factor and X_i_ is the expression of each factor (in our study represents gene expression). The optimal cutoff value was calculated using surv-cutpoint function. Then all the patients were divided into low-risk group (risk score lower than optimal cutoff) and high-risk group (risk score higher than optimal cutoff) based on the optimal cutoff value of Risk Score.

### Kaplan–Meier Survival Analysis

The overall survival rate of different groups was calculated by Kaplan–Meier method based on survival and survminer package of R software. The significance of the differences in survival rates between different groups was evaluated by the log-rank test.

### Proportion of Immune Cell Infiltration and Stromal–Immune Score

The relative proportion of 22 types of immune cell compositions was calculated by CIBERSORT software [22]. Based on the gene expression matrix, 547 genes preset by deconvolution algorithm was utilized to characterize the composition of infiltrating immune cells. Meanwhile, the sum of all the compositions of infiltrating immune cells must equal 1 for each sample. StromalScore, ImmuneScore, and ESTIMATEScore were calculated by the ESTIMATE package of R software.

### The Construction of Nomogram Prognostic Model

Nomogram was widely used to predict the prognosis of cancer [23]. Based on rms (https://CRAN.R-project.org/package=rms) package of R software, all independent prognostic factors identified by multivariate Cox regression analysis were included to construct a nomogram to predict the 1-, 3-, and 5-year survival for GC patients. The calibration curves were plotted to observe the Nomogram prediction probabilities against the actual probabilities. For each patient, 3 upward lines were drawn to confirm the points of each factor from the nomogram. After connecting these points and the “Total Points” axis, the downward lines of each factors were drawn to confirm probabilities of the 1-, 3-, and 5-year survival rate for GC patients.

## Results

### Identification of GC Metastasis-Related Genes

**Fig. 1:**
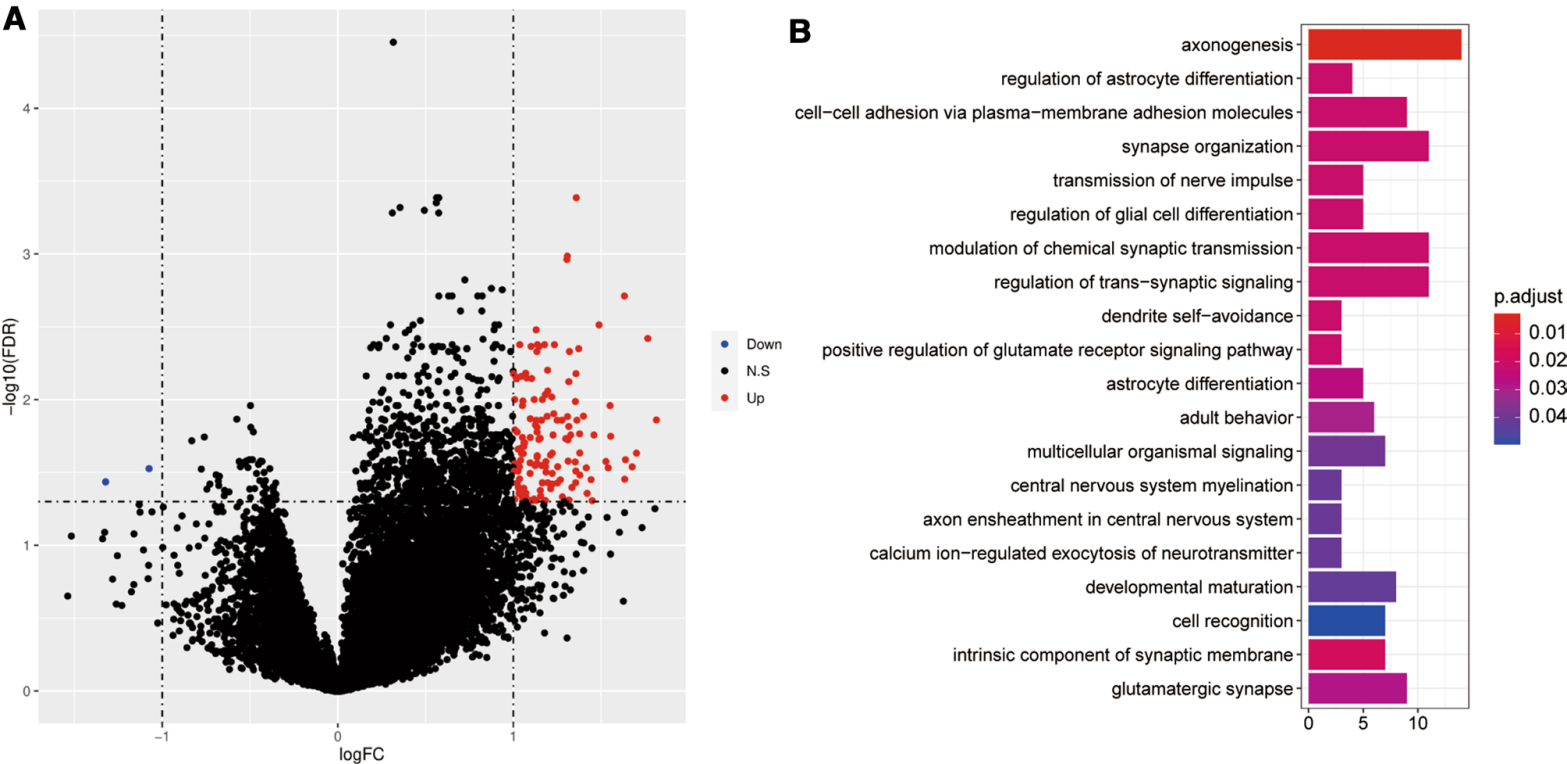
GC metastasis-related genes. **A** Volcano plot of DEGs. The horizontal axis represented the difference multiples after logarithmic conversion (Log_2_FC) and the vertical axis represented -log_10_ (FDR). Blue: down-regulated genes; red: up-regulated genes; **B** The top 20 enriched GO terms (Color figure online)

Basing on the data in TCGA cohort, the metastasis-related genes were analyzed between metastatic and non-metastatic GC samples. There were a total of 142 DEGs in metastatic GC samples compared with non-metastatic samples. We analyzed gene expression in metastatic and non-metastatic GC samples from the TCGA cohort and obtained 142 DEGs, including 140 up-regulated genes and 2 down-regulated genes (Fig. 1A). GO enrichment analysis showed that these 142 DEGs were mainly enriched in 31 GO terms, including 18 biological process terms (axonogenesis, regulation of astrocyte differentiation, cell–cell adhesion via plasma membrane adhesion molecules, synapse organization, transmission of nerve impulse, et al.), 5 cellular component terms (intrinsic component of synaptic membrane, glutamatergic synapse, cation channel complex, anchored component of plasma membrane, intrinsic component of postsynaptic membrane), and 8 molecular function terms (cell adhesion mediator activity, cell–cell adhesion mediator activity, sialic acid binding, clathrin binding, serine-type peptidase activity, et al.). Further KEGG pathway enrichment analysis showed that “cell adhesion molecules” was significantly enriched. The top 20 most significantly enriched Go terms are shown in Fig. 1B and detailed results of GO and KEGG enrichment analysis are listed in Table S1.

### The Risk Score Established by Eight Genes Could Predict the Prognosis for GC Patients

**Fig. 2:**
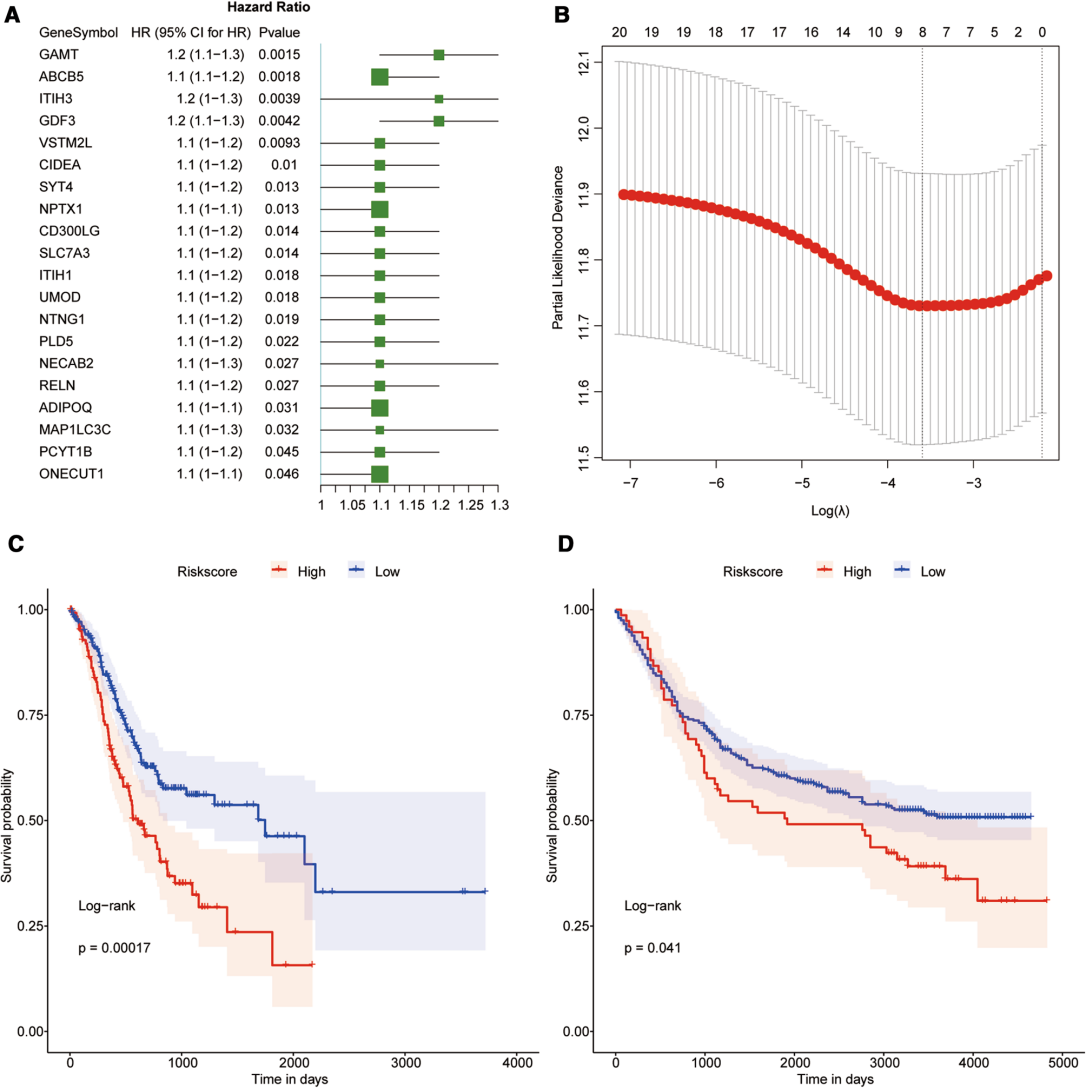
The construction of the GC prognosis model. **A** Forest map of 20 genes associated with prognosis of GC by univariate analysis. The HR was presented with its 95% confidence intervals (95% CI). **B** The LASSO regression model determined the tuning parameter (Lambda). The smallest value of partial likelihood deviance was the optimal Lambda value. **C** Kaplan–Meier curve of GC patients in TCGA database. **D** Kaplan–Meier curve of GC patients in GEO database

Taking the expression of 142 metastasis-related genes as a continuous variable, univariate Cox regression analysis was conducted and the Hazard ratio (HR) of each gene was calculated. HR > 1 indicated a risky gene with poor prognosis, while HR < 1 indicate a protective gene with favorable prognosis [24]. Subsequently, top 20 genes relating to GC prognosis were obtained (*p* value < 0.01) (Fig. 2A). Then LASSO Cox regression analysis was applied to further screen the optimal prognostic genes. According to the lambda-value of different genes, 8 genes were identified as the optimal genes (Fig. 2B), including GAMT, ABCB5, ITIH3, GDF3, VSTM2L, CIDEA, NPTX1, and UMOD. The z-score of gene expressions and the coefficient calculated by LASSO Cox regression were weighted to construct a Risk Score model as follows:

$$ {\text{Risk}}\,{\text{score}} = \left( {{\text{GAMT}}*0.0931646727} \right) + \left( {{\text{ABCB}}5*0.0615547169} \right) + \left( {{\text{ITIH}}3*0.0049653884} \right) + \left( {{\text{GDF}}3*0.0203733280} \right) + \left( {{\text{VSTM}}2{\text{L}}*0.0282159223} \right) + \left( {{\text{CIDEA}}*0.0128040421} \right) + \left( {{\text{NPTX}}1*0.0001032923} \right) + ({\text{UMOD}}*0.0361383549) .$$

basing on the expressions of the above 8 crucial genes, the risk score of each GC patient could be calculated, which would therefore predict the differential prognosis of patients. According to the optimal cutoff value of risk score, 0.0598, all GC samples from TCGA and GEO validation set were divided into high-risk group (risk score higher than optimal cutoff) and low-risk group (risk score lower than optimal cutoff). The results showed that GC samples from the high-risk group was associated with worse overall survival outcome compared with the low-risk group (Fig. 2C-D).

### Risk Score was an Independent Prognostic Factor

**Fig. 3:**
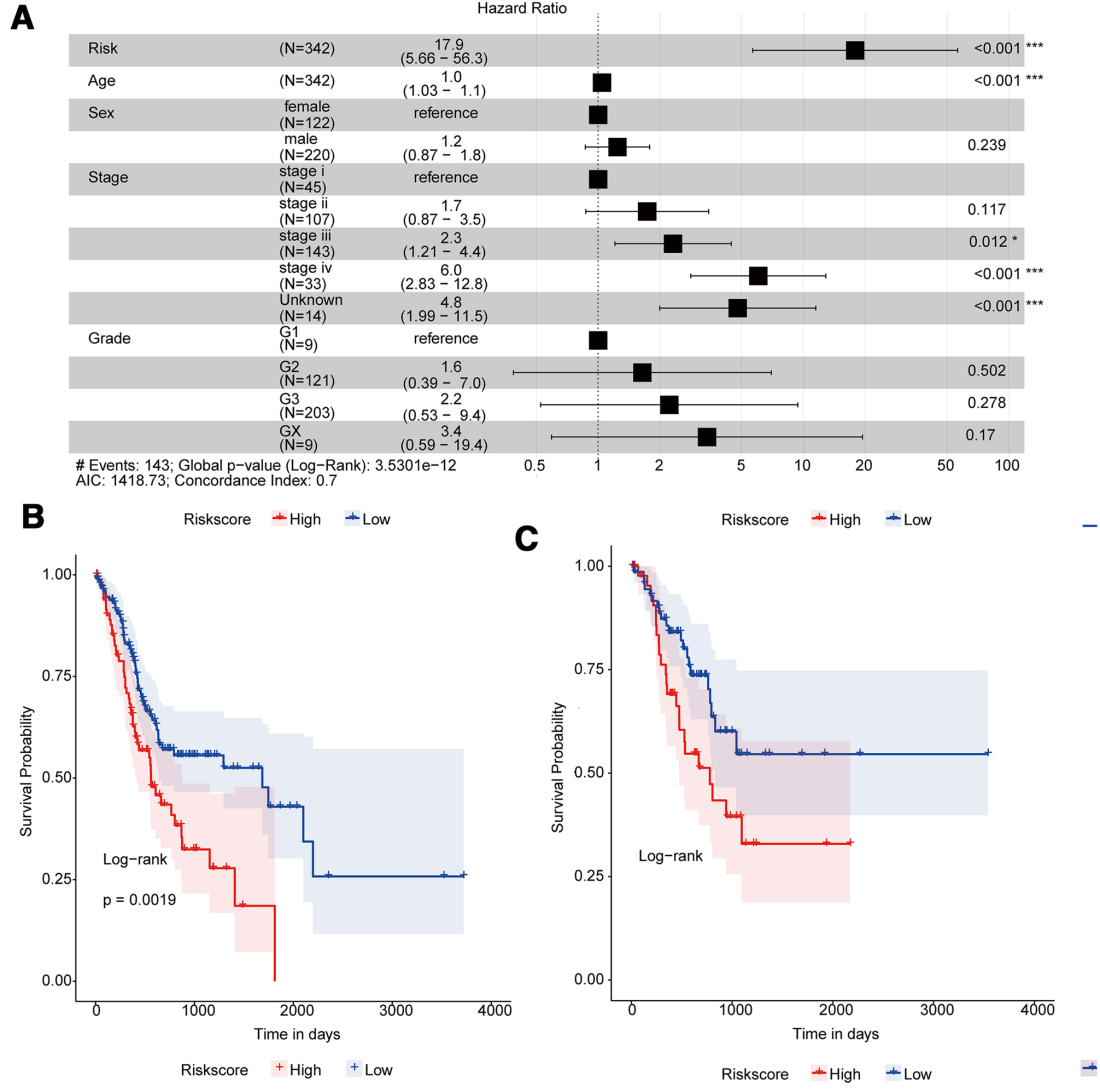

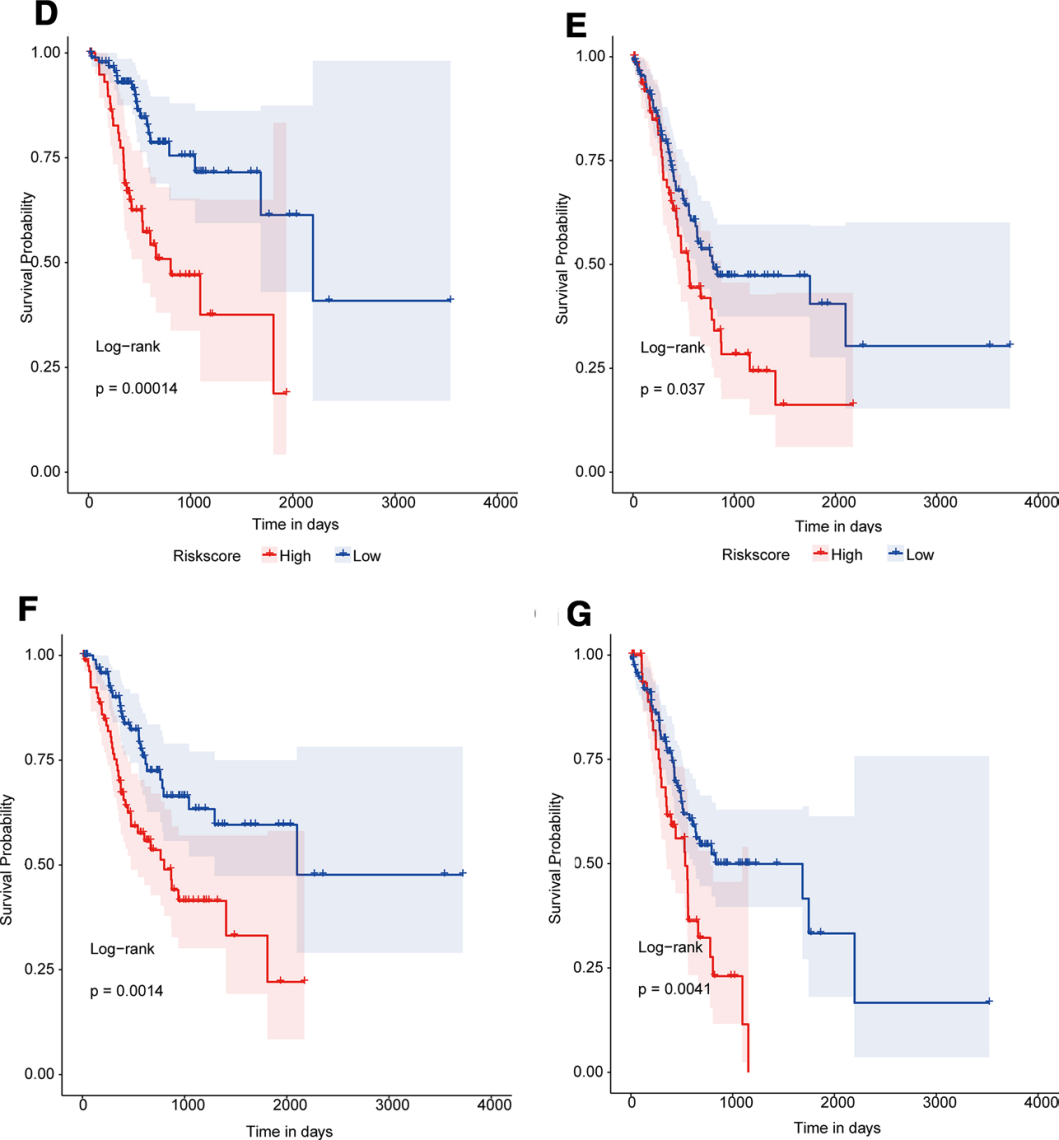
Stratified validation of the prognostic Risk Score model. The Risk Score was an independent prognostic indicator for GC patients. **A** Multivariate Cox regression analysis forest map. Compared with the reference sample, samples with HR > 1 represented a higher risk of death and HR < 1 represented a lower risk of death. **B**-**G** Kaplan–Meier survival curves of different groups. Different colors represented different groups. *p* value was calculated by log-rank test

To determine whether Risk Score was an independent prognostic factor, five factors including age, sex, stage, grade, and Risk Score were included in multivariate Cox regression analysis. As shown in Fig. 3A, Risk Score and stage were significantly associated with overall survival, high-Risk Score samples had a higher risk of death and were a poor prognostic factor (HR = 17.9, 95%*CI* 5.66–56.3, *p* < 0.001). In stratified analysis, the risk of death in the high-Risk Score group was significantly higher than that in the low-Risk Score group for all three clinical factors (sex: male and female, Fig. 3B-C; stage: early-stage I–II and advanced-stage III–IV, Fig. 3D-E; age: age ≥ 67 years old and age < 67 years old, Fig. 3F-G).

Overall, Risk Score based on GAMT, ABCB5, ITIH3, GDF3, VSTM2L, CIDEA, NPTX1, and UMOD could predict the prognosis for GC patients.

### Nomogram Model Could Predict the Prognosis for GC Patients

**Fig. 4:**
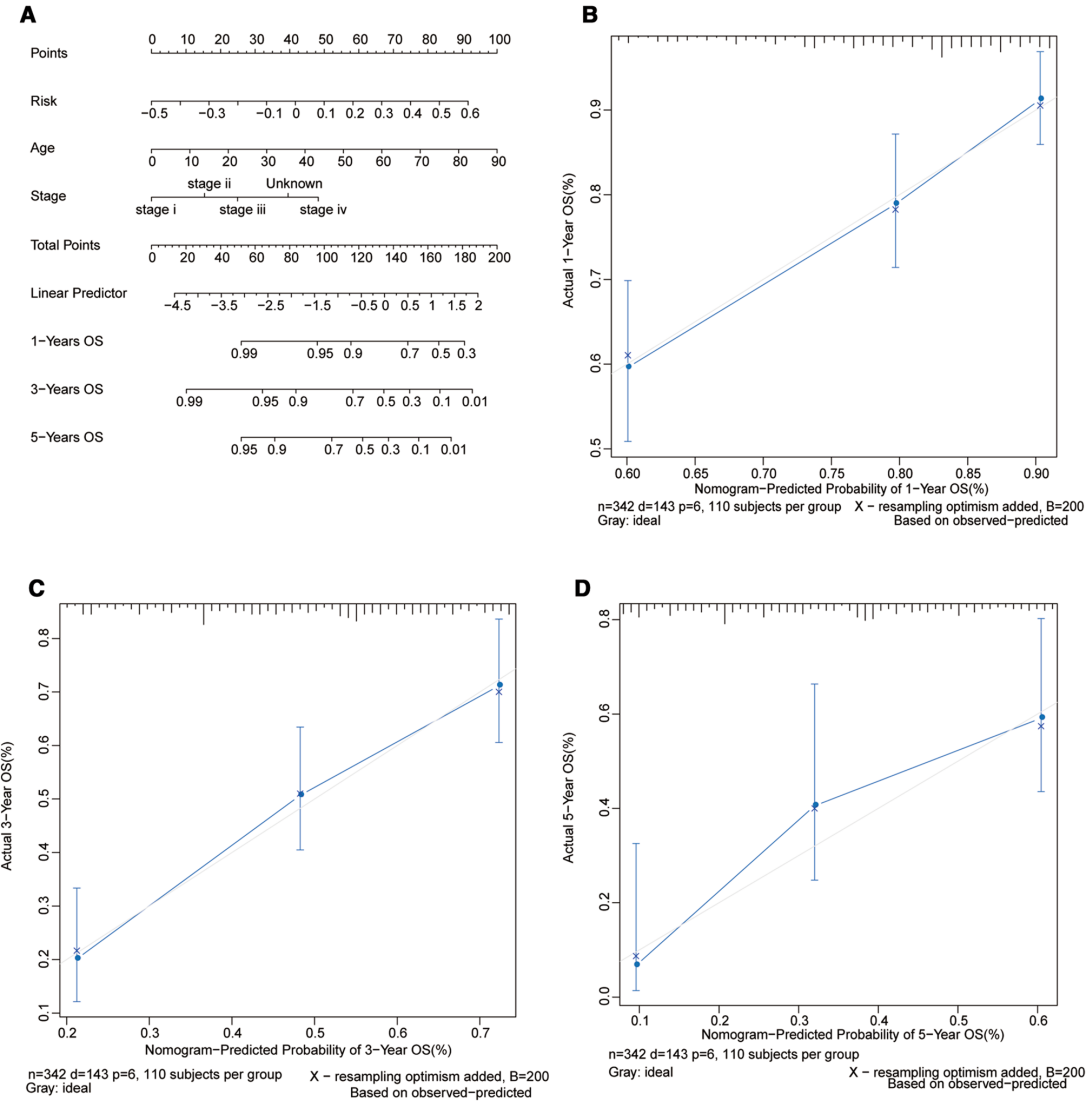
Nomogram could predict the survival probability of GC patients. **A** Nomogram for predicting the 1-, 3-, or 5-year overall survival time in GC patient. **B**-**D** Calibration curves of nomogram for predicting the 1-, 3-, or 5-year overall survival time in GC patients

The nomogram model was a valid model widely used to predict the prognosis of several cancers [25]. In this study, the nomogram model was constructed by age, stage, and Risk Score as three independent prognostic indicators (Fig. 4A). The results indicated that the corrected curves for 1-, 3-, and 5-year were close to the ideal curves (a 45-degree line with the slope of 1 through the origin of the coordinate axis) (Fig. 4B-D), suggesting that the predicted result of 1, 3, and 5 years were in good agreement with the actual results.

### Immune Cell Infiltration and Stromal–Immune Score in Differential Risk Score GC Patients

**Fig. 5:**
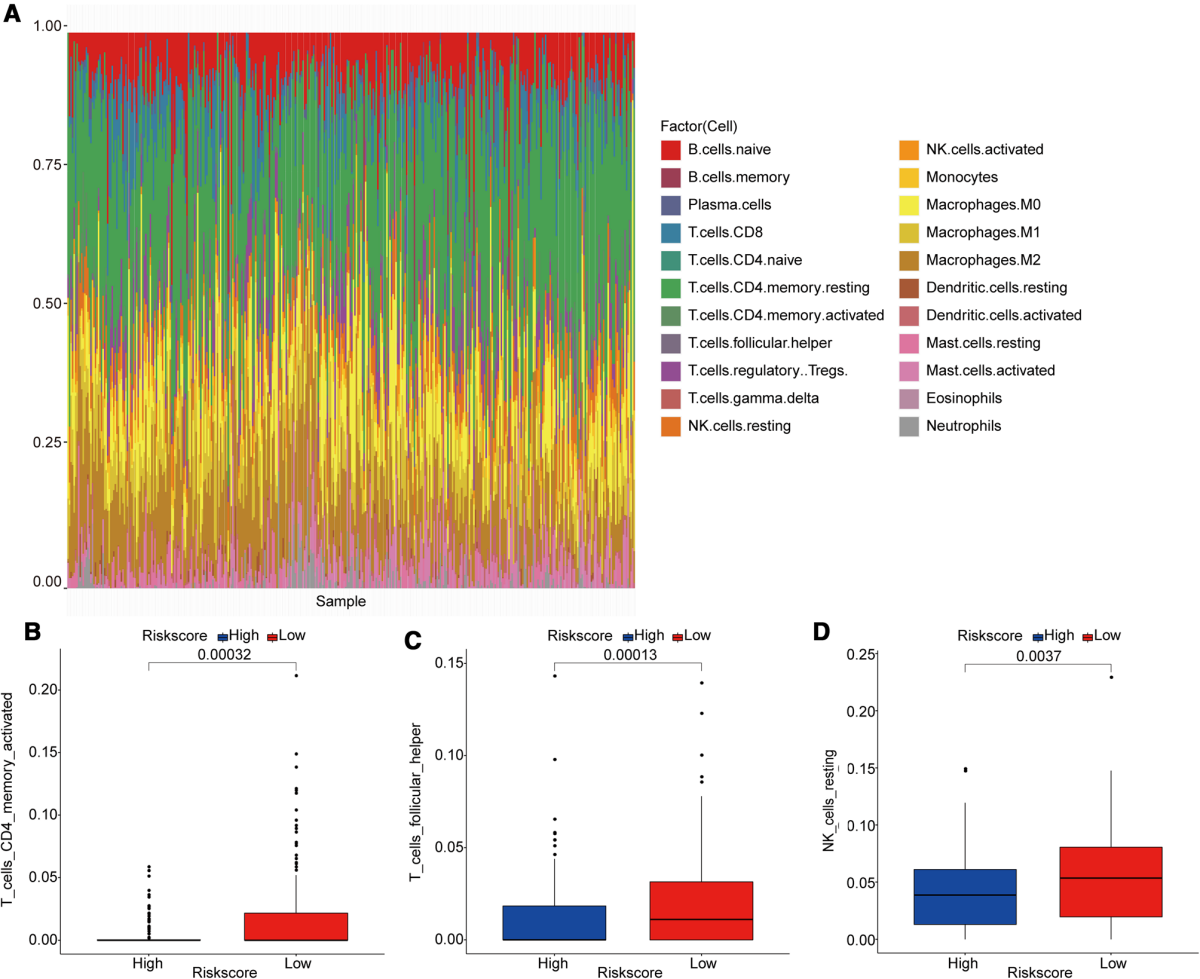

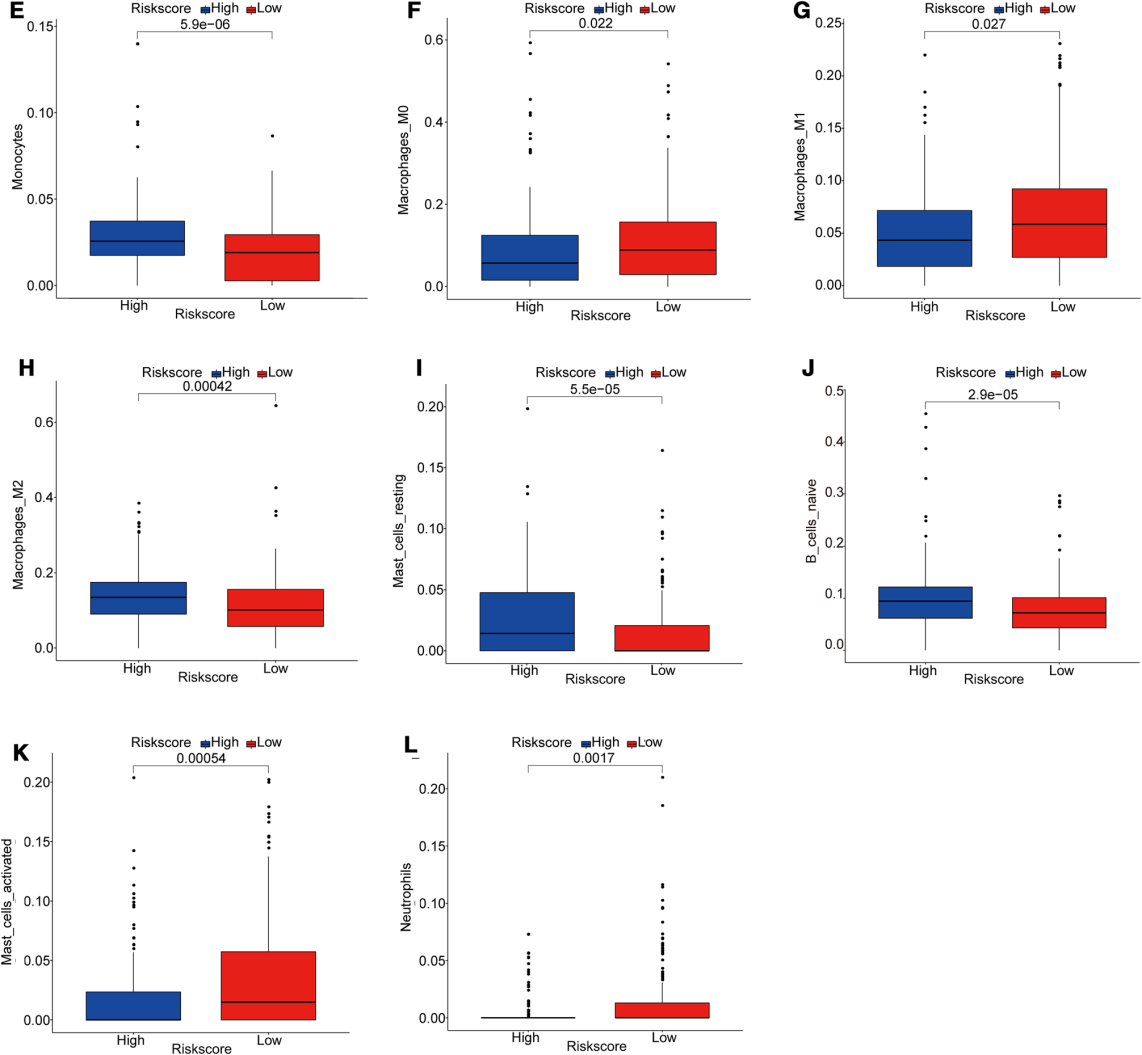
Immune cell infiltration in high- and low-risk GC patients. **A** The relative proportion of immune-infiltrating cells in all patients. **B**-**L** 11 types of immune cells with significantly different proportions of infiltration in the high- and low-risk groups. *p*-value was calculated by the Wilcoxon method

Finally, to further investigate the distinct immune landscape between high-risk and low-risk GC patients with differential survival, the immune cell infiltration and stromal–immune score were analyzed. Using the CIBERSORT method combined with the LM22 signature matrix, the relative infiltration levels of immune cells of GC patients in low-Risk Score group and high-Risk Score group were estimated. CIBERSORT employed a deconvolution algorithm to calculate the immune cell type and relative proportion via taking gene expression matrix as the input file. Then, the infiltration proportions of 22 types of immune cells in all 342 GC patients are summarized in Fig. 5A. Moreover, totally 11 types of immune cells, including B cells naïve, T cells CD4 memory activated, T cells follicular helper, NK cells resting, Monocytes, Macrophages M0, Macrophages M1, Macrophage M2, Mast cells resting, Mast cells activated, and Neutrophils, showed significant infiltrating differences between high- and low-risk GC groups (Fig. 5B-L). The changes in the proportion of tumor-infiltrating immune cells indicated the intrinsic differences in immune characteristics between high- and low-risk GC groups, which probably was an important aspect involving the differential prognosis.

**Fig. 6:**
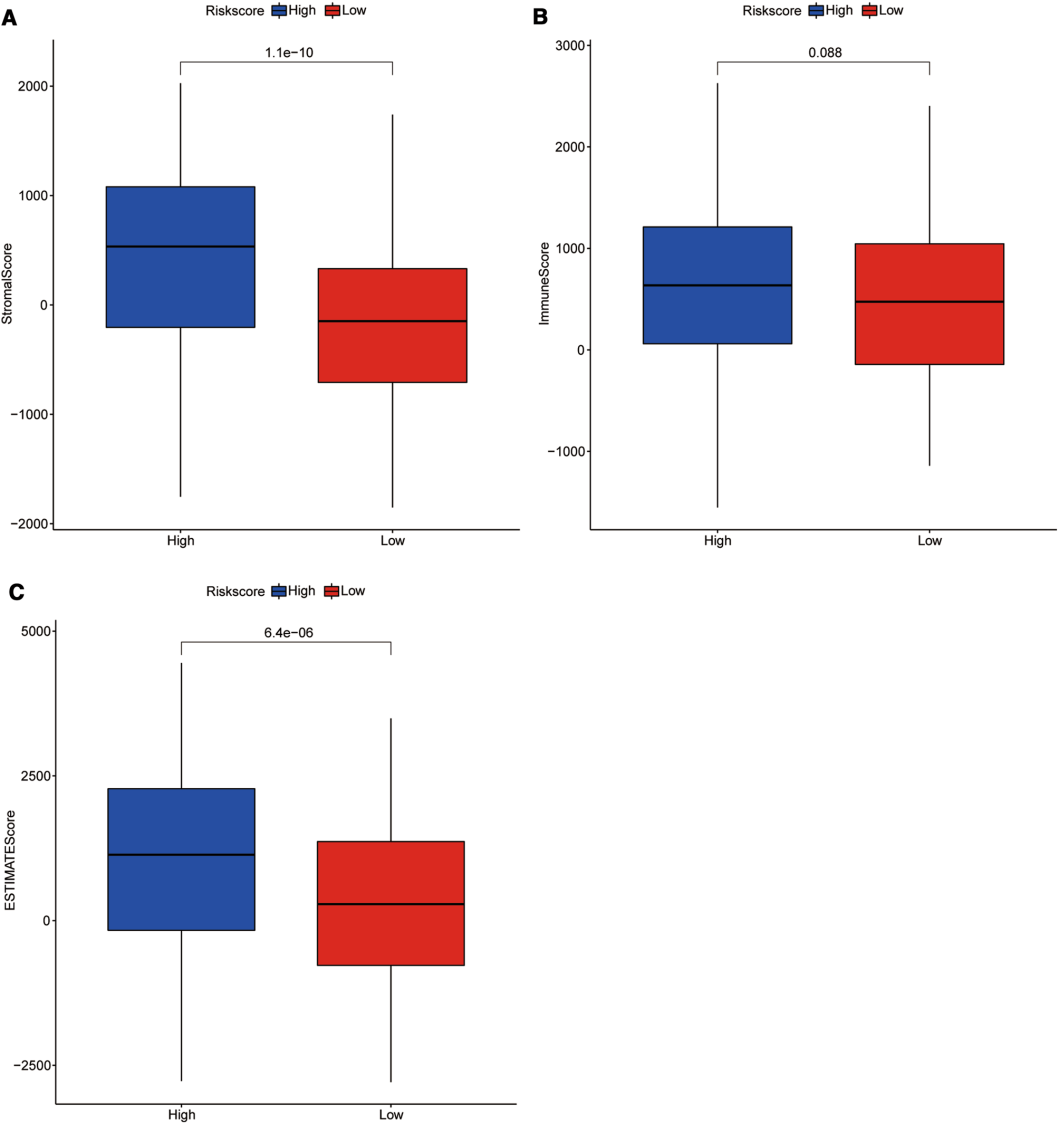
The StromalScore, ImmuneScore, and ESTIMATEScore of GC patients in high-risk and low-risk groups. **A** StromalScore. **B** ImmuneScore. **C**.ESTIMATEScore

In addition, StromalScore, ImmuneScore, and ESTIMATEScore were also estimated in high- and low-risk GC patients. ImmuneScore and StromalScore were used to evaluate the proportion of immune and stromal components in the tumor microenvironment (TME), respectively. EstimateScore was the sum of ImmuneScore and StromalScore, which represented the combined proportion of the two components in TME [26]. The higher the three scores, the more complex TME, and the complex TME, especially the immune microenvironment, were the main factor of poor prognosis [27]. Herein, our results suggested that the StromalScore, ImmuneScore, and ESTIMATEScore in high-Risk Score group were all higher than those in low-Risk Score group (Fig. 6A-C), further implying that the unfavorable immune microenvironment contributed to the poor prognosis of high-risk GC patients.

## Discussion

GC was one of the most common malignant tumors worldwide and the second leading cause of cancer death [28]. Since GC patients in the early stage were pauci- or asymptomatic, the vast majority of patients with GC were already in the advanced stage when a diagnosis was confirmed. For GC patients, cancer metastasis was often the main cause leading to disease aggravation and mortality [29]. Distant metastasis of GC occurs in three main ways: hematogenous metastasis, lymphatic metastasis, and disseminative [30, 31]. Many studies have confirmed that the prognosis of GC patients with gastric cancer was closely related to metastasis [32]. With the rapid development of genomics in recent years, an increasing number of metastasis-related genes were reported to be involved in the prognosis of GC patients. In the present study, totally 142 DEGs between metastatic and non-metastatic GC samples from TCGA were obtained, which were significantly enriched in 31 GO terms, such as axonogenesis, and one KEGG pathway, cell adhesion molecules. Combining all functional enrichment results, these metastasis-related DEGs showed a significant association with cell adhesion. Moreover, the decrease of cell adhesion has been proved to be related to the diffuse development of gastric cancer [33].

In this study, 8 optimal metastasis-related genes, comprising GAMT, ABCB5, ITIH3, GDF3, VSTM2L, CIDEA, NPTX1, and UMOD, were finally identified and a Risk Score model was built. Our results showed that both the TCGA database and GEO validation set with high-Risk Score group performed worse overall survival. Moreover, multivariate Cox regression analysis and the nomogram model indicated that Risk Score could serve as an independent prognostic factor for GC patients. Previous studies have reported the role of these genes in the progression of GC and other cancers. Adenosine triphosphate ATP-binding cassette (ABCB5), as an ABC transporter, has shown linked to the development of drug resistance in many kinds of cancers, such as melanoma, human retinoblastoma, and acute leukemia [34–36]. Accumulating evidence revealed that the inhibition of ABCB5 had an impact on the malignant potential of cancer cells both in vitro and in vivo [37]. ABCB5 has been reported as the hub genes correlated with the pathogenesis and prognosis of GC [38]. Inter-alpha (globulin) inhibitor H3 (ITIH3) was one of the five homologous heavy chains from the inter-alpha-trypsin inhibitors (ITI) family; Sébastien showed that ITIH3 expression increased cell attachment in vitro and indicated the antitumoral or antimetastatic properties of ITIH3 [39]. V-set and transmembrane domain-containing 2 like (VSTM2L) was expressed in multiple human tissues and has shown to be down-regulated in Helicobacter pylori-positive GC [40]. Moreover, the CpG island methylation phenotype (CIMP)-related gene signature comprising VSTM2L showed prognostic value in GC [41]. Neural pentraxin-1 precursor (NPTX1) was a member of the pentraxins family and was observed down-regulated in lung cancer, colon cancer, and pancreatic cancer [42–44]. Recent studies suggested that knockdown NPTX1 could suppress the migration, invasion, and adhesion abilities of GC cells and NPTX1 promoted GC metastasis via integrin/FaK signaling [45]. However, the detailed role of these genes in GC has not been fully revealed, which need to be further explored and demonstrated.

Recently, immune cells in tumor microenvironment were considered to be an important factor affecting prognosis [46, 47]. Therefore, the cell composition of the tumor microenvironment in GC samples were analyzed considering the crucial impacts of immune cells on tumor metastasis. Compared with low-risk GC patients, totally 11 types of immune cells were significantly differentially infiltrated in high-risk patients, which probably played more crucial roles in the distinct prognosis of GC patients. Among which, we noticed that the relative proportions of activated memory CD4 T cells and follicular helper T cells were significantly higher in low-risk patients. It has been documented recently that follicular helper T cells exerted an antitumor immune effect in a CD8 +-dependent manner [48], which could indirectly explain the better prognosis of low-risk patients. Moreover, M2 Macrophage infiltration proportion was significantly higher in high-risk patients with worse prognosis. Meanwhile, accumulating evidence has indicated that M2 Macrophages exerts various pro-tumoral activities, involving immunosuppression and the mediation of tumor metastasis process [49, 50]. Whereas, whether poor prognosis of high-risk GC patients was mainly caused by M2 Macrophage cannot be concluded in our present work, which deserved further investigation in future. What is not in doubt, the difference in the proportion of tumor-infiltrating immune cells indicated the intrinsic difference in the characteristics of tumor immune microenvironment between the two groups. Moreover, other stromal cells in the tumor microenvironment have also been confirmed to be involved in tumor progression, metastasis, and response to chemotherapy [51]. Panayiotou et al. have suggested that stromal cell ratio in the tumor microenvironment was of prognostic significance in patients with endometrial carcinoma [52]. In our study, the StromalScore, ImmuneScore, and ESTIMATEScore in the high-risk group were all significantly higher than the low-risk group, implying that the tumor microenvironment of the high-risk group was more complex. We suspected that the complex tumor microenvironment was one of the factors causing poor prognosis, but which cells play a dominant role and the mechanism remains to be further studied in future.

## Conclusion

In summary, the Risk Score model based on 8 metastasis-related genes, GAMT, ABCB5, ITIH3, GDF3, VSTM2L, CIDEA, NPTX1, and UMOD, has been established herein to distinguish prognosis of GC patients for the first time. The poor prognosis of GC patients with high-Risk Score might associate with the complex tumor microenvironments. Additionally, in our future work, the present prognostic model should be further validated in expanded clinical samples to strengthen the reliability, besides the underlying mechanism behind each gene remains to be elucidated in GC.

### Supplementary Information

Below is the link to the electronic supplementary material.

Supplementary file1 (XLSX 13 KB)—Detailed results of GO and KEGG enrichment analysis

## Data Availability

The datasets analyzed are available in The Cancer Genome Atlas database (TCGA, https://tcga-data.nci.nih.gov/tcga/)
